# Silica uptake by *Spartina*—evidence of multiple modes of accumulation from salt marshes around the world

**DOI:** 10.3389/fpls.2014.00186

**Published:** 2014-05-20

**Authors:** Joanna C. Carey, Robinson W. Fulweiler

**Affiliations:** ^1^Department of Earth and Environment, Boston UniversityBoston, MA, USA; ^2^Department of Biology, Boston UniversityBoston, MA, USA

**Keywords:** *Spartina*, salt marsh, silica, accumulation, grasses, wetland, ecosystem service

## Abstract

Silicon (Si) plays a critical role in plant functional ecology, protecting plants from multiple environmental stressors. While all terrestrial plants contain some Si, wetland grasses are frequently found to have the highest concentrations, although the mechanisms driving Si accumulation in wetland grasses remain in large part uncertain. For example, active Si accumulation is often assumed to be responsible for elevated Si concentrations found in wetland grasses. However, life stage and differences in Si availability in the surrounding environment also appear to be important variables controlling the Si concentrations of wetland grasses. Here we used original data from five North American salt marshes, as well as all known published literature values, to examine the primary drivers of Si accumulation in *Spartina*, a genus of prolific salt marsh grasses found worldwide. We found evidence of multiple modes of Si accumulation in *Spartina*, with passive accumulation observed in non-degraded marshes where *Spartina* was native, while rejective accumulation was found in regions where *Spartina* was invasive. Evidence of active accumulation was found in only one marsh where *Spartina* was native, but was also subjected to nutrient over-enrichment. We developed a conceptual model which hypothesizes that the mode of Si uptake by *Spartina* is dependent on local environmental factors and genetic origin, supporting the idea that plant species should be placed along a spectrum of Si accumulation. We hypothesize that *Spartina* exhibits previously unrecognized phenotypic plasticity with regard to Si accumulation, allowing these plants to respond to changes in marsh condition. These results provide new insight regarding how salt marsh ecosystems regulate Si exchange at the land-sea interface.

## Introduction

The role of nitrogen (N) and phosphorus (P) in plant productivity is well recognized by plant ecologists. Less well constrained is the importance of silicon (Si) in plant growth, maintenance, and fitness (Cooke and Leishman, 2011). In terrestrial ecosystems, Si is classified as a “quasi-essential” nutrient (Epstein, 2009) because it protects plants from a variety of stressors, including desiccation, heavy metal toxicity, and predation (Epstein, 1994; Hodson and Evans, 1995; Liang et al., 2007). Through their roots, plants take up dissolved silica (DSi) (SiO_2_) from the porewater, or soil solution, and transport it through the transpiration stream via the xylem. As water leaves the plant, Si becomes concentrated at transpiration termini and is deposited as biogenic Si (BSi). As such, plants often demonstrate increasing BSi concentrations with age or growth (Jones and Handreck, 1967; Epstein, 1994), due to continual precipitation of BSi at transpiration termini and the inability of plants to translocate Si once precipitated (Raven, 1983; Epstein, 1994). Although the majority of Si in plants is located at transpiration termini (e.g., leaves), Si is also found throughout other portions of plants, such as the roots, rhizomes, and stems (Struyf et al., 2005b; Querné et al., 2012; Carey and Fulweiler, 2013). The concentration of Si in plants is often higher than many macronutrients such as N and potassium (K). Si concentrations typically range from 0.1 to 10% by weight (by wt.) (Ma et al., 2001), the largest range of any element found in plants (Epstein, 1994).

The accumulation of Si in plants occurs via three possible modes of uptake: (1) active accumulation, where plants take up more Si than they would through water uptake alone, (2) passive accumulation, where plants have similar Si and water uptake rates, and (3) rejective or excluder accumulation, where Si uptake is slower than water uptake (Raven, 1983; Takahashi et al., 1990; Ma et al., 2001). These three possible modes of Si accumulation can be determined in several ways (Jones and Handreck, 1967; Takahashi et al., 1990; Ma et al., 2001). First, Si accumulation can be defined by the concentrations of Si in the aboveground plant tissue alone, where active accumulators typically have a dry weight of SiO_2_ >1% by wt., passive accumulators between 0.5 and 1% by wt., and excluders <0.5% by wt. (Ma et al., 2001; Street-Perrott and Barker, 2008; Hou et al., 2010; Carey and Fulweiler, 2012). The ratio of Si to calcium (Ca) is another means of determining the mode of Si accumulation, with Si:Ca ratios >1 indicating active accumulation, 0.5–1.0 indicating passive accumulation, and <0.5 indicating Si exclusion (Takahashi et al., 1990; Ma et al., 2001; Ma and Takahashi, 2002). Finally, the mode of Si accumulation can be determined based on the relationship between DSi porewater concentrations and aboveground biomass BSi concentrations (Jones and Handreck, 1967; De Bakker et al., 1999; Norris and Hackney, 1999; Ma et al., 2001). In this case, a positive relationship between porewater DSi concentrations and above ground BSi concentrations indicates passive accumulation, a negative relationship indicates active accumulation, and no relationship indicates rejective accumulation (Raven, 1983; De Bakker et al., 1999).

While all vegetation contains some Si (Epstein, 1994), grasses (*Poaceae*) and sedges (*Cyperaceae*) typically accumulate the most (Jones and Handreck, 1967; Raven, 1983; Ma and Takahashi, 2002), which has been attributed to active Si accumulation by these plants (Jones and Handreck, 1967; Raven, 1983). However, it remains uncertain whether all wetland grasses fall into this category of active accumulation. For example, evidence exists that *Spartina* grasses, which are one of the most prolific genus of salt marsh grasses worldwide, are passive accumulators (Hou et al., 2010; Querné et al., 2012) and possibly even rejector plants (De Bakker et al., 1999).

Understanding the mechanism responsible for Si accumulation by salt marsh plants is important for understanding the controls on Si exchange at the land-sea interface. Tidal marshes, are large reservoirs of Si (Struyf et al., 2005b; Carey and Fulweiler, 2013), and have been shown to play a critical role in regulating Si availability in adjacent estuarine waters (Struyf et al., 2005a; Jacobs et al., 2008; Vieillard et al., 2011). This has important consequences for marine trophic structure (Officer and Ryther, 1980), as diatoms, a dominant type of phytoplankton in temperate coastal waters, require as much Si as N on a molar basis to survive (Redfield et al., 1963). Because the Si found in marsh plants (i.e., BSi) dissolves several orders of magnitude faster than mineral silicates (Alexandre et al., 1997; Cornelis et al., 2010a), understanding how marsh grasses sequester Si is a key step for understanding how salt marshes regulate Si exchange in these dynamic ecotones.

Currently, controls on Si accumulation in salt marsh grasses are not well understood. For example, in addition to uncertainties regarding the mode of Si accumulation (i.e., active, passive, or rejective accumulation), the role of plant age or growth rate in controlling Si accumulation remains unclear (Querné et al., 2012). Although increasing BSi content with growth has been observed for *Spartina* (De Bakker et al., 1999; Norris and Hackney, 1999; Querné et al., 2012; Carey and Fulweiler, 2013) and several species of freshwater marsh plants (Struyf et al., 2005b), Hou et al. (2010) found no increase in *S. alterniflora* BSi concentrations over the growing season. The highly dynamic nature of salt marshes, which experience a wide range in nutrient availability, sediment type, and hydrologic conditions, could be responsible for the non-uniform BSi concentrations found in salt marsh plants. Furthermore, the amount of Si in the surrounding environment may also exert an important control over plant Si accumulation, especially in cases of passive Si accumulation (Struyf et al., 2005b). In these cases, increased Si availability in soil solution, or porewater, would result in higher Si accumulation in the plants.

The primary objective of this study was to determine the drivers of BSi accumulation in *Spartina*, one of the most common types of salt marsh grasses found worldwide. We hypothesize that differences in *Spartina* Si accumulation are driven by environmental conditions and plant origin (native vs. non-native). To test this hypothesis, we used original data from five New England (USA) salt marshes, as well as published literature values, to examine the mode of Si accumulation, and the role of plant growth and *in situ* Si availability (in sediment and porewater) in controlling BSi accumulation in *Spartina*. We then developed a conceptual model which suggests that differences in aboveground BSi concentrations are due not only to differences in Si availability in marshes, but also different modes of Si uptake, representing a previously unrecognized form of phenotypic plasticity in these plants.

## Materials and methods

**Figure 1:**
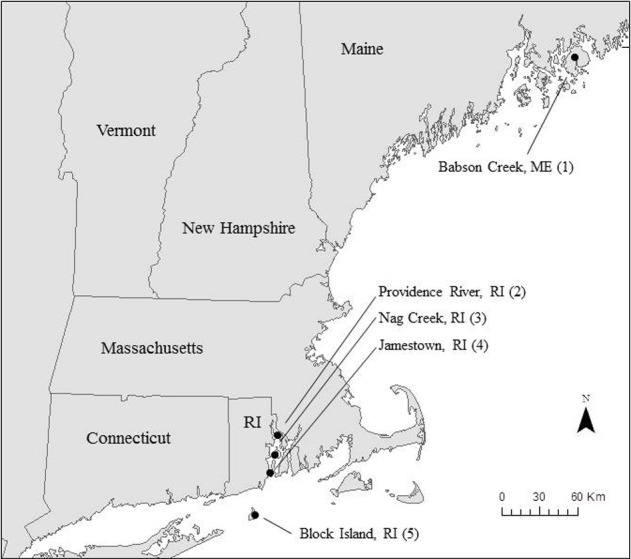
**Map of study region in New England, USA**.

We collected samples for Si accumulation at the height of growing season (spring) and during peak biomass (summer) in five marshes in New England, USA: a salt marsh in northern Maine (Site 1) and four Rhode Island (RI) salt marshes (Sites 2–5) (Figure 1, Table 1). All of the marshes displayed patterns of vegetation that are typical in New England marshes (Bricker-Urso et al., 1989; Wigand and Roman, 2012), with areas of “low marsh” that are inundated with tides twice a day consisting entirely of *Spartina alterniflora* grasses, while the areas of “high marsh” are inundated less frequently and are dominated by *S. patens* vegetation. Site 1 is a relatively undisturbed fringing salt marsh, located adjacent to Acadia National Park, draining an undeveloped watershed. The RI marshes cover a range of anthropogenic nutrient loadings and salinities (Table 1). Three of these marshes (Site 2–4) span the length of Narragansett Bay, from high to low nutrient inputs (DSi, N, P) (Figure 1, Table 1). Site 2 is a back-barrier marsh located on the east side of the Providence River Estuary, Site 3 is back-barrier marsh located on Prudence Island within the Narragansett Bay National Estuarine Research Reserve, and Site 4 is a fringing marsh located on the south side of Zeek's Creek in Jamestown, RI (Table 1). Site 5 is fringing marsh in Great Salt Pond located 21 km south of the coast of RI, on a relatively undisturbed island (Block Island) exposed to low-nutrient ocean water.

**Table 1:** **Site characteristics of estuaries adjacent to the salt marshes included in analysis**.

**Study and/or site name**	**Estuary**	**Salinity (ppt)**	**Bulk density (g cm^−3^**	**DIN (μM)**	**DIP (μM)**	**DSi (μM)**	**Data source(s)**
**This Study**							
1 (Babson Creek, ME)	Sommes Sound, USA	13.9 ± 2.7	0.70 ± 0.08	4.8 ± 0.2	0.23 ± <0.01	48 ± 6	Carey and Fulweiler, unpublished
2 (Little Mussachuck Creek)	Providence River Estuary, USA	27.7 ± 0.12	0.24 ± 0.04	4.5 ± 0.7	1.5 ± 0.11	19.6 ± 1.8	Krumholz, 2012 (nutrients)
3 (Nag Creek West)	Narragansett Bay, USA	30.0 ± 0.06	0.26 ± 0.03	1.5 ± 0.2	1.0 ± 0.08	16.7 ± 1.7	Krumholz, 2012 (nutrients)
4 (Zeek's Creek)	Narragansett Bay, USA	30.2 ± 0.12	0.25 ± 0.02	1.4 ± 0.1	0.73 ± 0.05	12.4 ± 1.0	Krumholz, 2012 (nutrients)
5 (Block Island)	Great Salt Pond, USA	29.2 ± 0.12	1.15 ± 0.04	6.2 ± 0.9	0.67 ± 0.11	na	URI Watershed Watch (www.uri.edu/ce/wq/ww) (nutrients)
Carey and Fulweiler, 2013 High-N (Apponaug)	Narragansett Bay, USA	30.1 ± 0.1	0.2 ± <0.01	22.3 ± 6	0.4 ± 0.12	45.6 ± 2.7	Carey and Fulweiler, unpublished
Querné et al., 2012	Bay of Brest, France	32–35	na	<5.6	<0.1	<1	Dauchez et al., 1991 (salinity)
							Ragueneau et al., 1994 (nutrients)
De Bakker et al., 1999	Oosterschelde, The Netherlands	25	0.3–0.6	3.5^*^^,^ ^**^	0.57^**^	4.5^**^	Nienhuis and Smaal, 1994 (nutrients)
							Oenema and DeLaune, 1988 (bulk density)
Norris and Hackney, 1999	Cape Fear River Estuary, USA	18.6	0.84 ± 0.07	9.3^**^	0.23^**^	63.8^**^	Freese, 2003, PhD dissertation (bulk density)
							Mallin et al., 1997 (nutrients, salinity)
Hou et al., 2010	Yangtze River Estuary, China	>20	1.3	22^**^	1.2^**^	28.6^**^	Yang et al., 2008 (Bulk density)
							Chai et al., 2009 (nutrients, salinity)

Unless otherwise noted, all values summer averages ± standard error.

* Does not include NH_4^+^_,

** annual average.

We measured net Si accumulation in the aboveground vegetation, sediment, and porewater from the *S. alterniflora* zone at all sites. At sites 1 and 3, we also collected samples for aboveground vegetation, sediment, and porewater from the *S. patens* zone of the marshes. Sediment cores from sites 2–5 were also sieved for roots and rhizomes in order to analyze BSi content in belowground vegetation. In all cases, triplicate measurements of each of the parameters were sampled. Sampling was paired, so that in all cases where we sampled aboveground vegetation, samples for sediment, belowground vegetation, and porewater were also collected. Sediment cores and porewater were sampled from within the 0.25 m^2^ plot where aboveground vegetation was collected. The same marshes were sampled during each season, but not the exact same 0.25 m^2^ plot within each marsh, as our sampling was destructive (e.g., removal of grass and sediment from marsh). All samples from each individual marsh were collected on a single day. The triplicate values from each individual marsh were averaged for each season (spring and summer). These average values for spring and summer were treated as distinct value for all analyses.

We harvested above ground vegetation from three randomly chosen 0.25 m^2^ plots per sampling event. Vegetation was washed thoroughly with deionized water and dried at 70°C for 48 h before it was ground using a Wiley Mill. We collected sediment cores using PVC corers (30 cm long, 5 cm diameter). We divided our cores into four sections—the top 1, 1–10, 10–20, and 20–30 cm. In order to calculate bulk density (mass of material per unit volume), we dried each core section at 70°C until it reached a constant mass over two consecutive days of weighing. Next, the cores were wet-sieved (0.5 mm sieve) to isolate roots and rhizomes, which were subsequently washed, dried, weighed, and analyzed for BSi concentrations. We collected triplicate porewater samples using porewater peepers, a passive sampling technique (Templer et al., 1998). We followed methods detailed in Carey and Fulweiler (2013), where 20-mL scintillation vials are pre-filled with deionized water and capped with a permeable membrane in order to allow the exchange of ions. Each polyvinyl chloride (PVC) peeper contained 10 vertically-stacked vials placed 3 cm apart. Peepers were deployed vertically in the sediment for 2–3 weeks, after which time the water was filtered using a 60-mL polypropylene syringe through a 0.45 micron nitrocellulose filter. The timing of peeper deployment always corresponded with collected of the other samples (e.g., sediment cores, aboveground vegetation). Porewater samples were stored in polyethylene bottles and kept in a cool dark place until they were analyzed for DSi. At sites 2–5, we also measured salinity and pH of all porewater samples.

We quantified sediment amorphous Si (ASi) content [which includes both BSi and pedogenic Si (Cornelis et al., 2010a)] and biomass BSi concentrations using the wet chemical alkaline extraction in 1% Na_2_CO_3_ solution (Demaster, 1981). Biomass was digested for 4 h. Sediment was digested for 5 h, with sub-samples taken at hours 3 and 4 in order to calculate a mineral correction (Demaster, 1981; Conley and Schelske, 2001). We used a Seal AA3 flow injection analyzer and the molybdenum blue colorimetric method (Strickland and Parsons, 1968) to measure DSi concentrations. We routinely compared our standards to Hach external standards and they were always within 4% of the expected value.

In addition to our collected data from the five marshes in New England, we gathered all known published studies reporting aboveground plant tissue *Spartina* BSi concentrations and either productivity (biomass per unit area) and/or porewater DSi concentrations. In total, we located five studies that fit these characteristics (Table 2). The five studies presented data from salt marshes on three continents—Europe (i.e., the Netherlands, France), North America (i.e., New England and Southeast, USA), and Asia (i.e., China). We then identified general site characteristics of each site (e.g., bulk density and N availability) for comparison (Table 1).

**Table 2:** **Studies included in this analysis**.

**Study**	**Location**	**Species**	**Variables reported**	**Type**
This study	New England, USA	*S. patens, S. alterniflora*	BSi, DSi, Productivity	Native
Carey and Fulweiler, 2013	New England, USA	*S. patens, S. alterniflora*	BSi, DSi, Productivity	Native
Norris and Hackney, 1999	North Carolina, USA	*S. alterniflora*	BSi, DSi, Productivity	Native
Querné et al., 2012	Bay of Brest, France	*S. alterniflora*	BSi, DSi, Productivity	Invasive
Hou et al., 2010	China	*S. alterniflora*	B Si, Productivity	Invasive
De Bakker et al., 1999	Netherlands	*S. anglica*	BSi, DSi	Invasive

Variables reported: DSi indicates porewater DSi (SiO_2_) concentrations, BSi indicates aboveground tissue plant BSi concentrations. Productivity indicates biomass per unit area.

In order to test the hypothesis that primary productivity or *in-situ* Si availability was driving aboveground BSi concentrations in *Spartina*, we ran correlation analysis between BSi concentrations in aboveground material and the following variables: primary productivity [a proxy for plant age for perennial grasses, which reach maximum productivity in August and senesces in the fall (Wigand et al., 2004)], ASi concentrations in the sediment, and DSi concentrations in porewater. We also ran simple linear regression and used the least squares method to estimate model parameters, with *Spartina* BSi concentrations as the independent variable in all cases. In addition to examining all data together to see if broad trends in *Spartina* BSi behavior were apparent, we grouped our data by species and region to determine any differences related to taxonomy or location. We used the slopes of the regressions between *Spartina* BSi concentrations and porewater DSi concentrations to identify the mode of Si accumulation in these plants, with strong positive slopes indicating passive accumulation, negative slopes indicating active accumulation and flat slopes indicating rejective accumulation. Comparisons across sites were determined using a One-Way ANOVA and “multcompare” command in Matlab. All statistics were done using Matlab using α of 0.05 as the threshold for significance. All BSi concentrations reported as percent SiO_2_ by dry weight (dry wt.).

## Results

### Si content of New England *S. alterniflora*

A large range of aboveground BSi concentrations in *S. alterniflora* were found in the marshes, and concentrations typically increased throughout the growing season (Table 3). The minimum concentration observed was 0.24% by wt. (Site 5 in spring) and the maximum concentration measured was 1.04% by wt. (Site 2 in summer). These values align well with values reported in a meta-analysis by Hodson et al. (2005) and show that compared to other plant species in the order Poales, *Spartina* has relatively low Si concentrations.

**Table 3:** **BSi concentrations in aboveground and belowground biomass, and ASi concentrations in sediment at each site**.

**Site**	**Aboveground (% BSi)**		**Belowground Biomass (% BSi)**		**Sediment (% ASi)**
	**Spring**	**Summer**	**Roots**	**Rhizomes**	
*S. alterniflora*					
1	0.53 ± 0.06	0.95 ± 0.11	na	na	2.12 ± 0.49
2	0.48 ± 0.13	0.96 ± 0.05	1.04 ± 0.15	0.22 ± 0.04	3.76 ± 0.39
3	0.43 ± 0.07	0.60 ± 0.09	1.04 ± 0.17	0.24 ± 0.04	3.19 ± 0.44
4	0.30 ± 0.01	0.71 ± 0.13	1.39 ± 0.29	0.35 ± 0.07	2.83 ± 0.51
5	0.26 ± 0.02	0.45 ± 0.02	0.66 ± 0.11	0.23 ± 0.05	0.35 ± 0.08
*S. patens*					
1	1.01 ± 0.22	0.89 ± 0.14	na	na	2.16 ± 0.35
3	0.29 ± 0.07	0.31 ± 0.02	0.36 ± 0.04	0.13 ± 0.02	1.31 ± 0.27

Sediment values reported as ASi, as measurements include both biogenic and pedogenic Si. Where seasonal differences were observed (aboveground biomass only), we report both seasons separately. Otherwise, values represent average ± standard error across both seasons.

Sediment ASi concentrations were almost always higher in the top 1 cm of the sediment compared to the depths of 1–30 cm (except Site 3 in spring) (Table S1). We found no significant differences in ASi concentrations seasonally or among sites, except for Site 5, which had ASi concentrations (0.35 ± 0.07%) an order of magnitude lower than all other sites in this study (Avg. 2.28 ± 0.47% by wt.) (Table 3, Table S1). Similar to the sediment ASi, the top one cm of the roots always had higher BSi content than the deeper depths (Table S2). Across all sites and both seasons, root BSi values ranged from a minimum of 0.18% observed at Site 5 in the summer to maximum value of 4.83% observed at Site 4 in summer. The average root BSi concentration across all sites and seasons was 1.03 ± 0.31%. Consistent with earlier observations in the same region (Carey and Fulweiler, 2013), the rhizomes always had significantly less BSi than the roots (Avg. 0.26 ± 0.03% by wt.) (Table S2). We found no seasonal or site related patterns to BSi concentrations in roots and rhizomes (Table S2).

Porewater DSi concentrations were higher during the summer compared to the spring and porewater values from Site 1 were typically higher than from the other marshes (Table 4, Table S3). Porewater concentrations ranged from 0 μM (top 1 cm at Site 2 in spring) to 384 μM (depth of 27 cm depth at Site 1 in summer) (Table S3). Across marshes, porewater pH was significantly (*p* < 0.01) different during both spring and summer seasons. For example, during the summer the pH at Sites 4 and 5 was significantly (*p* < 0.01) lower compared to Site 2.

**Table 4:** **pH, salinity, and DSi concentrations (average ± standard error) measured in top 30 cm of porewater at each marsh site in this study**.

**site**	**Spring**			**Summer**		
	**pH**	**Salinity (ppt)**	**DSi (μM)**	**pH**	**Salinity (ppt)**	**DSi (μM)**
*S. alterniflora*						
1	na	na	181.5 ± 33.3	na	na	239.5 ± 32
2	7.47 ± 0.09	24.0 ± 0.3	115.2 ± 18.3	7.07 ± 0.09	25.7 ± 0.4	156.3 ± 7.0
3	7.09 ± 0.02	29.6 ± 0.1	83.3 ± 4.1	7.22 ± 0.08	31.2 ± 0.4	121.7 ± 5.3
4	7.32 ± 0.07	30.1 ± 0.9	25.1 ± 3.3	6.42 ± 0.05	31.3 ± 0.4	55.4 ± 6.6
5	7.45 ± 0.03	30.0 ± 0.1	5.4 ± 1.1	6.60 ± 0.34	32.6 ± 0.3	55.1 ± 6.9
*S. patens*						
1	na	na	204.1 ± 39.4	na	na	153.0 ± 18
3	7.11 ± 0.07	28.3 ± 0.9	42.8 ± 5.6	7.3 ± 0.08	32.13 ± 1.1	49.5 ± 1.6

### Si content of New England *S. patens*

At two of the marshes in this study (Sites 1 and 3) we analyzed samples for Si accumulation in *S. patens*, or the high marsh platform zone of the marsh. Overall, Site 1 had higher Si accumulation than Site 3. For example, aboveground BSi concentrations of *S. patens* were roughly three times higher at Site 1 (Table 3), ranging from 0.58 to 1.25% by wt. (Site 2) and 0.21 to 0.43% (Site 3). Likewise, sediment ASi concentrations were also higher at Site 1 (Avg 2.16 ± 0.35% by wt.) than at Site 3 (Avg 1.31 ± 0.27% by wt.) (Table 3, Table S1). Similar to *S. alterniflora*, the ASi concentrations in the top one cm of sediment were always higher than the deeper layers of the core (except for summer Site 1 sample where 10–20 cm depth had slightly higher concentration) (Table S1). BSi in the roots and rhizomes in *S. patens* ranged from 0.08 to 0.96% and similar to *S. alterniflora*, BSi concentrations of the rhizomes (0.13 ± 0.02%) were much lower than in the roots (0.36 ± 0.04%) (Table S1).

Similar to other portions of the marsh budget, porewater concentrations were an order of magnitude higher at Site 1 than at Site 3 on all occasions (Table 4, Table S3). Average porewater salinity (30.2 ppt) and pH (7.21) at Site 3 under *S. patens* vegetation was similar to the values measured under *S. alterniflora* (Table 4).

### BSi accumulation as a function of productivity

**Figure 2:**
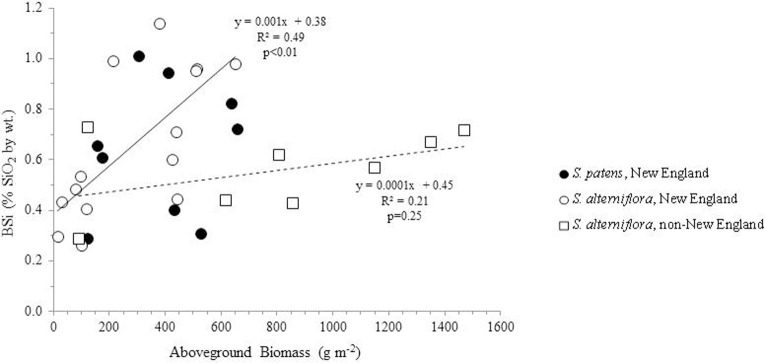
**BSi concentration in aboveground *Spartina* (*S. alterniflora* and *S. patens*) as a function of productivity**. Using the entire dataset, no relationships were found in the data (*R*^2^ = 0.05, *n* = 31). Similarly, no relationships between the variables were found in *S. patens* (filled circles) (*R*^2^ = 0.02, *n* = 9) or non-New England *S. alterniflora* (*R*^2^ = 0.21, *n* = 8) (dashed trend line). However, New England *S. alterniflora* BSi concentrations showed a significant (*p* < 0.01, *n* = 14) positive relationship with productivity (*R*^2^ = 0.49) (solid trendline). In total these values represent nine marshes: three non-New England marshes (China, France, North Carolina, USA) and six New England marshes. New England data represent average values of triplicate field measurements, with separate points for spring and summer seasons. Individual points representing non-New England marshes either represent distinct seasons (Norris and Hackney, 1999; Hou et al., 2010) or marshes (Querné et al., 2012).

Using all available data from our analysis and the literature, we found a no correlation between aboveground productivity (mass per unit area) and BSi concentrations (*n* = 31, *R* = 0.22, *p* = 0.23) (Figure 2). We next subdivided the entire data by species and by location in order to determine if regional or taxonomic differences resulted in distinct relationships between productivity and BSi accumulation in these plants. However, this continued to result in no significant relationships between productivity and BSi concentrations in most situations: *S. patens* (*n* = 9, *R* = 0.14, *p* = 0.72) and the non-New England *S. alterniflora* (*n* = 8, *R* = 0.46, *P* = 0.25). In fact, we only found the expected positive relationship between productivity and BSi concentration in New England *S. alterniflora* (*n* = 14, *R* = 0.70, *p* <0.01) (this study, Carey and Fulweiler, 2013) (Figure 2). Although the ways in which BSi concentrations vary as a function of productivity is not uniform across sites, we found that all marshes, regardless of location, begin the growing season with a fairly consistent amount of BSi in *S. alterniflora* tissue (0.38 to 0.45% by wt.) (y-intercept, Figure 2). However, after the initial growing period, BSi accumulation diverges.

### BSi accumulation as a function of *in situ* Si availability

In order to test our hypothesis that *in situ* Si availability impacts BSi accumulation in aboveground plant tissue, we wanted to determine whether elevated sediment ASi concentrations or DSi in the porewater was associated with higher concentrations of aboveground BSi concentrations. Sediment ASi data was only available in conjunction with aboveground BSi concentrations for our original data, and for Carey and Fulweiler (2013). We found a strong correlation (*n* = 22, *R* = 0.60, *p* < 0.01) between sediment availability and BSi content (Table 3).

We found several studies, in addition to our own original data presented here, where porewater DSi concentrations are presented in conjunction with aboveground BSi concentrations (Table 2). Similar to the relationship between aboveground BSi content and productivity, examining all the data together resulted in no relationship between the concentrations of BSi in aboveground biomass and the concentration of DSi in the porewater (*n* = 43, *R* = −0.20, *p* = 0.21). We then subdivided the data by region and/or species in order to determine if there were differences based on taxonomy or origin. In doing so, different and yet, distinct relationships between porewater DSi and biomass BSi concentrations became apparent.

#### North american marshes

**Figure 3:**
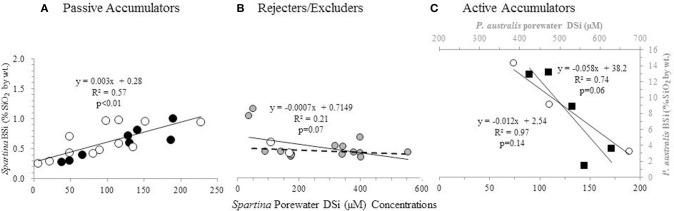
**BSi concentration in aboveground biomass as a function of porewater DSi concentrations**. *S. patens* (black circles), *S. anglica* (filled gray circles), and *S. alterniflora* (open white circles), *P. australis* (black squares). Dashed line in **(B)** indicates the regression ignoring the two *S. anglica* outliers. Values in **(A,C)** represent average values from triplicate field measurements in top 20 cm of sediment, with spring and summer values plotted individually. Values in **(A)** represent values from five New England salt marshes. Values in **(B)** represent ten marshes in Oosterschelde, and the two marshes in Bay of Brest (Querné et al., 2012). Values in **(C)** represent values from one marsh in New England (*S. alterniflora* data) and one marsh in Belgium [*P. australis* data from Struyf et al. (2005b)].

A positive correlation between *Spartina* BSi concentrations and porewater DSi concentrations (*n* = 20, *R* = 0.77, *p* < 0.01) (Figure 3A) was observed when all the data from New England was grouped together [excluding the data from the degraded “high-N” marsh described by Carey and Fulweiler (2013), which is discussed below]. In examining this regional grouping by species, we found a similar positive relationship for both *S. patens* (*n* = 8, *R* = 0.93, *p* < 0.01) and *S. alterniflora* (*n* = 12, *R* = 0.72, *p* < 0.01). However, data from one New England marsh [*S. alterniflora* at the N-enriched marsh (“high-N” site) presented by Carey and Fulweiler, 2013] showed a distinctly different pattern, as we observed a negative relationship (*n* = 3, *R* = −0.98, *p* = 0.14) between porewater DSi concentrations and BSi concentrations (Figure 3C).

Besides the data from the New England salt marshes, Norris, and Hackney completed a study of net Si accumulation in a mid-Atlantic marsh (North Carolina, USA). This study also observed a negative relationship between porewater DSi concentrations and *S. alterniflora* BSi concentrations (*n* = 3, *R* = −0.94, *p* = 0.22). However, unlike the pattern observed in the high-N marsh described by Carey and Fulweiler (2013), the data from this North Carolina marsh showed the opposite relationship with time, as over the course of growing season plant BSi concentrations decreased, while porewater concentrations increased (Figure S1).

#### European marshes

We observed a weak relationship between plant BSi and porewater DSi concentrations (*n* = 18, *R* = −0.45, *p* = 0.07) in the data from the European marshes (De Bakker et al., 1999; Querné et al., 2012). This weak negative relationship was primarily driven by two data points of low DSi porewater concentration (Figure 3B). Removing these two points (both of which are *S. anglica*) resulted in no relationship between those two variables (*n* = 16, *R* = 0.07, *p* = 0.80).

## Discussion

It is not uncommon for a single species of plant to contain different amounts of Si (Hodson et al., 2005), but such variability is typically thought to be due to differences in Si availability in the surrounding environment or growth rates (Norris and Hackney, 1999; Struyf et al., 2005b; Guntzer et al., 2012; Querné et al., 2012; Carey and Fulweiler, 2013). With regard to *Spartina*, productivity does not appear to be a major control over Si accumulation. Despite commencing the growing season with a relatively uniform amount of BSi, the relationship between productivity and aboveground BSi accumulation diverges at higher levels of productivity (Figure 1). This divergence indicates that productivity does not control BSi accumulation in the same manner in all *Spartina* species.

Differences in transpiration rates have been shown to be responsible for different levels of Si accumulation in plant leaves (Cornelis et al., 2010b; Guntzer et al., 2012). As such, the lack of relationship between productivity and BSi concentrations may be due to variable transpiration rates among salt marhes and/or water availability across these marshes, supporting the idea that productivity is not a good proxy for water use (Hessini et al., 2009). In addition to potential differences in water availability across sites, our results indicate that variable amounts of bio-available Si present in the system and different modes of Si accumulation by the plants are the likely drivers of this pattern.

### Passive Si accumulation in New England marshes

In the New England marshes, higher ASi availability in the sediments is associated with higher BSi accumulation in the plants. This correlation is likely due to the soluble nature of ASi, which is considered a biologically available form of Si, having dissolution rates orders of magnitudes faster than mineral silicates (Struyf and Conley, 2012). New England salt marshes have some of the highest recorded sediment ASi concentrations (Carey and Fulweiler, 2013), which may explain why the BSi concentrations observed at several of these sites is higher than those found elsewhere (Figure 1).

Similar to the relationship observed with sediment ASi, higher porewater DSi concentrations are associated with higher aboveground BSi concentrations in all New England data, except in a highly degraded marsh (high-N marsh, Carey and Fulweiler, 2013) (discussed below) (Figure 3A). The positive relationship indicates that in the non-degraded New England marshes, *Spartina* passively accumulates Si, as more Si in the surrounding environment is associated with more Si in aboveground plant tissue. Thus, in this situation of passive accumulation, *in situ* Si availability in both the sediment and porewater are important controls Si accumulation in *Spartina*.

### Rejective Si accumulation in European marshes

Unlike the strong positive and negative relationships we observed between plant BSi concentrations and porewater DSi concentrations in the New England marsh, we found no relationship between these two variables in the European marshes (Figure 3B). The lack of relationship between porewater DSi and plant BSi concentrations points toward *Spartina* in these systems behaving as a Si excluder, particularly at higher Si concentrations. In fact, De Bakker et al. (1999) also came to the same conclusion about their data, and suggested that *S. anglica* may reject Si from its tissue via a barrier on the outer surface of the roots, similar to legumes (Raven, 1983).

The extensive reviews by Jones and Handreck (1967) and Raven (1983) point toward rejector plants accumulating 5–60% of the DSi that is in the transpired water, with the remainder being left in the porewater. Further, at higher soil solution concentrations, plants will reject a larger portion of DSi (Raven, 1983; Cornelis et al., 2010b), which we also find evidence for with our flat regression line between aboveground BSi content and porewater DSi concentrations (Figure 3B). The exact mechanisms driving Si rejection in plants are not yet well understood, but it has been suggested that low Si accumulating plants have either a defective or non-existent Si transporter from cortical cells into the xylem (Ma and Yamaji, 2008).

Our designation of a Si excluder to *S. alterniflora* in the French salt marsh disagrees from the original interpretation of the data (Querné et al., 2012). While Querné et al. (2012) do point out the lack of relationship between DSi porewater and plant BSi concentrations, they assign *S. alterniflora* to the passive accumulator category based on the dry weight of BSi in the aboveground tissue alone. While this is a common practice when porewater DSi data is unavailable (Hou et al., 2010; Carey and Fulweiler, 2012), it ignores differences in transpiration or *in situ* Si availability. Thus, only defining Si accumulation by aboveground BSi concentration data may be misleading when passive Si accumulators are exposed to unusually high or low porewater concentrations, as one could incorrectly assign passive accumulators active or rejective status based on dry weight alone (Liang et al., 2005). Based on our analysis of the invasive *Spartina* from Europe, and the range of porewater concentrations observed in salt marshes, we are now skeptical at using the dry weight of BSi alone in quantifying mode of Si accumulation in marsh grasses.

### Active Si accumulation in degraded marsh

Unlike all other data from New England, we observed a negative relationship between porewater DSi availability and aboveground BSi content in the highly degraded salt marsh described in Carey and Fulweiler (2013) (Figure 3C). This negative relationship provides evidence of active Si accumulation, with the plants sequestering Si in high enough proportions to drive down porewater concentrations (Raven, 1983). As described by Raven (1983) and Jones and Handreck (1967), active Si accumulation results in the decline of DSi concentrations in the “bathing medium” as the plant takes up DSi relatively faster than it takes up water. Agricultural studies of active transport in rice, a known active Si accumulator, has identified two ATP-fuelled Si transporters (Ma and Yamaji, 2008), which transports Si through the membranes. Moreover, we consistently observed aboveground BSi concentrations >1% by wt., the threshold concentration shown to indicate active accumulation (Ma et al., 2001; Street-Perrott and Barker, 2008; Carey and Fulweiler, 2012; Querné et al., 2012).

In order to provide further support for our interpretation that the negative relationship between porewater DSi concentrations and aboveground BSi concentrations indicates active Si accumulation, we looked to Struyf et al. (2005b), who examined these two variables in a Western European tidal freshwater marsh dominated by *Phragmites australis*. *P. australis* is a known active Si accumulator, allowing us to examine the relationship between plant BSi and porewater DSi concentrations in a marsh experiencing active accumulation. Using the data presented by Struyf et al. (2005b), we found that *P. australis* also exhibits a strong negative relationship between porewater DSi concentrations and aboveground BSi concentrations (*n* = 5, *R* = −0.86, *p* = 0.06) (Figure 3C). The similar relationship between porewater and aboveground BSi concentrations in *P. australis* and the *S. alterniflora* in the degraded high N marsh provides additional evidence of active Si accumulation in the degraded marsh.

This site where evidence of active accumulation is observed is a high-nutrient, degraded salt marsh located downstream of an urban watershed (Wigand et al., 2003; Carey and Fulweiler, 2013). In fact, the N loading here is so high that the marsh is now P-limited, with a summer average molar DIN/DIP ratio in the inundating tidal creek water of 53 (Table 1). While it is possible that external factors are resulting in the declining porewater concentrations over time at this marsh, the fact that we do not observe this trend in the four nearby marshes indicates that the degraded, high N nature of the marsh is playing a role in the distinct behavior observed in *Spartina* here.

The results of one study, which focused on Si accumulation in a North Carolina (USA) salt marsh (Norris and Hackney, 1999), did not fit the criteria for any of the three modes of uptake. During our re-analysis of those data we observed a strong negative relationship between porewater DSi concentration and plant BSi concentration. While this trend initially indicates active accumulation, further examination of the change through time reveals that throughout the course of growing season, plant BSi concentrations decreased, while porewater concentrations increased (Figure S1). This is the opposite temporal relationship that one would find with active accumulation and does not fit the expected trend for passive or rejective accumulation either which signals a departure from any of the three traditional modes of uptake. The observations from this marsh call into question whether or not some plants, under certain conditions, can translocate Si.

### Environmental stress and phenotypic plasticity as drivers of Si accumulation?

In examining the site characteristics of these marshes, we identify several variables that may drive the different modes of Si accumulation within marsh plants. The marshes studied here span a range of conditions, from nutrient over-enriched to relatively undisturbed, exposing these grasses to variable amounts of stress. Studies of abiotic stress on plant BSi concentrations report mixed results. Querné et al. (2012) found no plant response upon exposure to several types of stress, while Schoelynck et al. (2012) found increased Si accumulation in macrophytes exposed to intense water currents. A recent study of a degraded, “high-N” marsh found significantly more Si accumulation in several components of the marsh budget compared to a lower nutrient marsh (Carey and Fulweiler, 2013), which was hypothesized to be due in part to the stress induced by high air temperatures and low amounts of rainfall. We now propose that this degraded salt marsh is actively accumulating Si, which we suggest is a defense mechanism to ameliorate environmental stress.

In addition to marsh condition, genetic origin represents another variable across sites. *S. alterniflora* is native to the Atlantic and Gulf of Mexico coasts of North America, but invasive in many areas of the world, including the Pacific coast of North America, Europe, and Asia. Similarly, *S. anglica* is invasive in Western Europe. Invasive species in general have been shown to exhibit higher phenotypic plasticity than non-invasive species (Davidson et al., 2011). Phenotypic plasticity refers to the ability of organisms to change their observable traits in response to environmental conditions (Schlichting, 1986; Davidson et al., 2011). This phenomenon may be responsible for the different patterns in Si accumulation by salt marsh plants. Invasive species, particularly *S. alterniflora*, have been shown to exhibit higher phenotypic plasticity than the native plants (Zhao et al., 2010), which may explain the contrasting Si behavior by the same genus of plants. In particular, a recent study in China found the invasive *S. alterniflora* to show a higher degree of morphological growth, and biomass allocation plasticity in association with N availability compared to the native grass (Zhao et al., 2010). Our data suggests that genetic origin is another factor driving the differences in BSi accumulation in *Spartina* across sites. While phenotypic plasticity has been demonstrated for N, to our knowledge this is the first study to suggest a phenoplastic response of *Spartina* to Si.

### A conceptual model describing drivers of *Spartina* Si uptake

**Figure 4:**
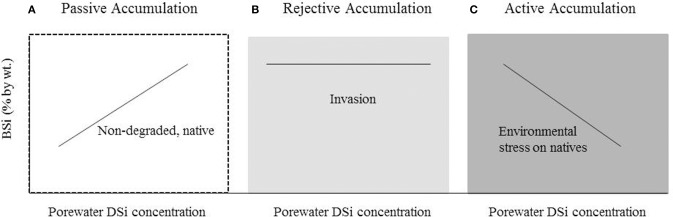
**Conceptual model hypothesizing how three modes of Si uptake in *Spartina* are related to site characteristics of those marshes, following the trends found in Figure 3. (A)** Passive accumulation where native *Spartina* is found in non-degraded marshes, **(B)** Rejective accumulation where *Spartina* is invasive, **(C)** Active accumulation where native *Spartina* is exposed to environmental stress.

Based upon the available data and our analysis above it is clear that wetland grasses cannot be grouped into one mode of Si accumulation. Rather we hypothesize that different Si accumulation modes are based on site-specific environmental conditions and genetic origin. To demonstrate this hypothesis we developed a conceptual model depicting the different modes of Si uptake by *Spartina* (Figure 4), which is based on the different slopes we observed in our linear regressions between porewater DSi availability and aboveground BSi concentrations (Figure 3). As illustrated in our model, we propose that *Spartina* passively accumulates Si in non-degraded marshes where the species are native, such as the case in the non-impaired New England marshes included in this study (Figures 3A, 4A). Further, we suggest that *Spartina* behaves as Si excluders/rejectors in situations where the plant is invasive (Figures 3B, 4B). Finally, we hypothesize that active Si accumulation by the native *Spartina* occurs when the plants are exposed to highly stressful, degraded conditions (Figures 3C, 4C).

We present our conceptual model as a hypothesis which must be tested in future salt marsh research. Our hypothesis takes into account all known studies where *Spartina* BSi concentrations are reported alongside either porewater DSi concentrations or sediment ASi concentrations, but other confounding factors (e.g., diatom uptake, interaction with mineral silicates, and transport processes) also play a role in salt marsh Si dynamics. Thus, future work should focus on controlled field observations and laboratory studies which account for confounding variables as a means of testing the conceptual model that we put forth here. The recent recognition that gene expression can control Si accumulation in rice (Ma et al., 2006, 2007) highlights an exciting avenue of potential research for salt marsh grasses, such as *Spartina*.

While this is the first study to provide evidence of variable modes of accumulation in *Spartina*, Liang et al. (2005, 2007) observed both active and passive Si accumulation within several of the same species of plants (e.g., cucumber, rice, maize, sunflower, and wax gourd). Rather than the absolute categorization of “active” accumulator to grasses, the mode of Si accumulation within *Spartina* is variable depending on site-specific conditions. This interpretation aligns with the previous suggestion that plant species should be placed along a spectrum of accumulation, rather than categorized as having only one method of accumulation (Cooke and Leishman, 2011), an idea supported by recent work at the molecular-level of plant Si biochemistry (Ma et al., 2006, 2007; Ma and Yamaji, 2008).

### Active Si accumulation and a loss of ecosystem service?

Tidal wetlands are important regulators of Si fluxes to adjacent estuarine systems (Struyf et al., 2005a; Jacobs et al., 2008; Vieillard et al., 2011), often supplying DSi necessary for diatom growth in coastal systems (Anderson et al., 2002; Danielsson et al., 2008). A recent mass balance of Si fluxes in a New England salt marsh found the marsh to be a “point” source of DSi to the adjacent estuary, primarily due to drainage of Si-rich porewater during the ebbing tide (Vieillard et al., 2011). We observed a 60% decline in marsh porewater concentration over the growing season (spring through fall) in the marsh experiencing active Si accumulation. In turn, we hypothesize that the presence of active Si accumulators in marshes may diminish the export of DSi to downstream coastal systems by reducing DSi porewater concentrations. While the BSi sequestered in the plants will eventually be remineralized, this may occur after the plant has been exported from the marsh. Even in the case that BSi is remineralized *in situ*, it will ultimately shift the timing of DSi availability in the system. A shift to active Si accumulation by marsh grasses exposed to degraded conditions may represent a previously unrecognized way that coastal nutrient enrichment can impact estuarine ecology in North America.

Likewise *P. australis* is an invasive species throughout North American salt marshes (Chambers et al., 1999). We hypothesize that a shift in vegetation type from that of passively accumulating *Spartina* to actively accumulating *P. australis* could result in lower marsh porewater DSi concentrations. Such lower porewater DSi concentrations could reduce the ability for salt marshes to serve as sources of DSi to adjacent estuaries. In turn, we predict that invasion of *P. australis* to North American salt marshes may alter the timing and magnitude of Si exchange in coastal systems, highlighting a potentially unrealized consequence of this invasion to coastal ecosystems.

Tidal wetlands have the ability to control Si availability in coastal systems, which has important consequences for phytoplankton species composition. Here we present evidence that *Spartina* grasses may shift their mode of Si accumulation based on local environmental conditions and genetic origin. Recognition that these wetland grasses accumulate Si differently depending on site-specific characteristics provides new insight regarding the role these grasses play in Si dynamics at this borderland between land and sea.

### Conflict of interest statement

The authors declare that the research was conducted in the absence of any commercial or financial relationships that could be construed as a potential conflict of interest.
