# Supplementary material for: Silica uptake by Spartina—evidence of multiple modes of accumulation from salt marshes around the world
Source: Front Plant Sci. 2014 May 20;5:186. doi: 10.3389/fpls.2014.00186 (PMC4033131; doi:10.3389/fpls.2014.00186)
Supplement: Supplementary file 1 [file DataSheet1.DOCX]

**Supplementary Information** for Carey and Fulweiler (*submitted*) “Silica uptake by *Spartina* – evidence of multiple modes of accumulation from salt marshes around the world”

**Supplementary Figures**

Figure S1: BSi concentration in aboveground biomass as a function of porewater DSi concentrations from Norris and Hackney (1999) (gray circles) and Carey and Fulweiler (2013) (nutrient over-enriched, ‘high-N’, site). Arrows indicate increase through time. The high-N site (Carey and Fulweiler 2013) exhibits a signal of active Si accumulation (increasing BSi concentration, declining porewater DSi concentrations over time), while the Norris and Hackney (1999) data show the opposite trend, with decreasing BSi concentrations throughout the growing season (average of mature and new species in April, July, and September). This pattern follows none of the three modes of Si uptake.

**Supplementary Tables**

Table S1. Sediment ASi concentration profile for each site during the spring and summer.

| **Sediment ASi (%SiO_2_ by wt)** | | | | | |
| --- | --- | --- | --- | --- | --- |
|  | Spring | |  | Summer | |
| Depth (cm) | Average | Std Error |  | Average | Std Error |
|  | *S. alterniflora* | | | | |
| **Site 1** |  |  |  |  |  |
| 0-1 | 4.83 | 0.66 |  | 3.33 | 1.26 |
| 1-10 | 3.11 | 0.46 |  | 2.18 | 0.42 |
| 10-20 | 0.95 | 0.06 |  | 1.35 | 0.21 |
| 20-30 | 0.70 | 0.19 |  | 1.09 | 0.37 |
|  |  |  |  |  |  |
| **Site 2** |  |  |  |  |  |
| 0-1 | 5.49 | 0.98 |  | 5.62 | 1.62 |
| 1-10 | 1.98 | 0.72 |  | 1.38 | 0.43 |
| 10-20 | 2.85 | 0.53 |  | 3.78 | 0.59 |
| 20-30 | 4.18 | 0.58 |  | 4.81 | 0.53 |
|  |  |  |  |  |  |
| **Site 3** |  |  |  |  |  |
| 0-1 | 3.33 | 1.57 |  | 6.01 | 0.71 |
| 1-10 | 3.39 | 1.61 |  | 2.38 | 1.02 |
| 10-20 | 1.50 | 0.55 |  | 2.52 | 0.35 |
| 20-30 | 4.63 | 1.29 |  | 1.74 | 0.85 |
|  |  |  |  |  |  |
| **Site 4** |  |  |  |  |  |
| 0-1 | 4.34 | 0.81 |  | 7.13 | 2.73 |
| 1-10 | 0.78 | 0.10 |  | 2.83 | 0.75 |
| 10-20 | 1.59 | 0.16 |  | 2.43 | 0.48 |
| 20-30 | 1.76 | 0.27 |  | 1.77 | 0.10 |
|  |  |  |  |  |  |
| **Site 5** |  |  |  |  |  |
| 0-1 | 1.15 | 0.39 |  | 0.24 | 0.06 |
| 1-10 | 0.55 | 0.16 |  | 0.23 | 0.01 |
| 10-20 | 0.21 | 0.03 |  | 0.16 | 0.03 |
| 20-30 | 0.13 | 0.03 |  | 0.17 | 0.00 |
|  |  |  |  |  |  |
|  | *S. patens* | | | | |
| **Site 1** |  |  |  |  |  |
| 0-1 | 4.60 | 0.83 |  | 3.18 | 0.36 |
| 1-10 | 3.17 | 0.45 |  | 2.70 | 0.40 |
| 10-20 | 2.13 | 0.59 |  | 3.13 | 0.23 |
| 20-30 | 0.68 | 0.11 |  | 1.37 | 0.11 |
|  |  |  |  |  |  |
| **Site 3** |  |  |  |  |  |
| 0-1 | 2.19 | 1.26 |  | 3.46 | 1.02 |
| 1-10 | 1.15 | 0.50 |  | 0.80 | 0.28 |
| 10-20 | 0.62 | 0.23 |  | 1.00 | 0.21 |
| 20-30 | 0.40 | 0.26 |  | 0.47 | 0.17 |

Table S2: Belowground vegetation BSi concentration profile for each site during the spring and summer.

| **Root BSi (%SiO_2_ by wt.)** | | | | | |  | **Rhizome BSi (%SiO_2_ by wt)** | |
| --- | --- | --- | --- | --- | --- | --- | --- | --- |
|  | Spring | |  | Summer | |  | Spring | Summer |
| Depth (cm) | Average | Std Error |  | Average | Std Error |  |  |  |
|  | *S. alterniflora* | | | | | | | |
| **Site 2** |  |  |  |  |  |  |  |  |
| 0-1 | 1.36 | 0.35 |  | 2.45 | 0.49 |  | NA | NA |
| 1-10 | 0.54 | 0.11 |  | 0.59 | 0.07 |  | 0.13 | 0.21 |
| 10-20 | 0.75 | 0.19 |  | 0.59 | 0.04 |  | 0.15 | 0.31 |
| 20-30 | 0.97 | 0.30 |  | 1.05 | 0.37 |  | 0.14 | 0.39 |
|  |  |  |  |  |  |  |  |  |
| **Site 3** |  |  |  |  |  |  |  |  |
| 0-1 | 1.90 | 0.88 |  | 1.45 | 0.27 |  | 0.49 | NA |
| 1-10 | 1.47 | 0.81 |  | 0.43 | 0.05 |  | 0.16 | 0.26 |
| 10-20 | 0.83 | 0.25 |  | 0.80 | 0.27 |  | 0.18 | 0.17 |
| 20-30 | 0.93 | 0.38 |  | 0.50 | 0.24 |  | 0.25 | 0.19 |
|  |  |  |  |  |  |  |  |  |
| **Site 4** |  |  |  |  |  |  |  |  |
| 0-1 | 3.66 | 1.08 |  | 2.40 | 1.21 |  | 0.73 | NA |
| 1-10 | 1.46 | 0.59 |  | 0.87 | 0.23 |  | 0.17 | 0.20 |
| 10-20 | 1.01 | 0.19 |  | 0.53 | 0.12 |  | 0.35 | 0.42 |
| 20-30 | 0.55 | 0.19 |  | 0.61 | 0.21 |  | 0.36 | 0.22 |
|  |  |  |  |  |  |  |  |  |
| **Site 5** |  |  |  |  |  |  |  |  |
| 0-1 | 1.58 | 0.36 |  | 0.39 | 0.06 |  | 0.39 | NA |
| 1-10 | 1.31 | 0.26 |  | 0.39 | 0.01 |  | 0.46 | 0.19 |
| 10-20 | 0.71 | 0.14 |  | 0.39 | 0.07 |  | 0.21 | 0.13 |
| 20-30 | 0.32 | 0.03 |  | 0.19 | 0.01 |  | 0.11 | 0.12 |
|  |  |  |  |  |  |  |  |  |
|  | *S. patens* | | | | | | | |
| **Site 3** |  |  |  |  |  |  |  |  |
| 0-1 | 0.56 | 0.21 |  | 0.34 | 0.04 |  | 0.11 | NA |
| 1-10 | 0.25 | 0.03 |  | 0.38 | 0.16 |  | 0.08 | 0.10 |
| 10-20 | 0.33 | 0.12 |  | 0.54 | 0.12 |  | 0.08 | 0.20 |
| 20-30 | 0.16 | 0.05 |  | 0.34 | 0.13 |  | 0.16 | 0.16 |

Table S3: Porewater DSi concentration profiles for each site during spring and summer.

| **Porewater DSi (μM)** | | | | | | | |
| --- | --- | --- | --- | --- | --- | --- | --- |
|  | *S. alterniflora* | | | | | *S. patens* | |
|  | Spring | | | | | | |
| Depth (cm) | Site 1 | Site 2 | Site 3 | Site 4 | Site 5 | Site 1 | Site 3 |
| 3 | 58.0 | 0.2 | 50.2 | 3.3 | 6.5 | 145.9 | 8.8 |
| 6 | 131.0 | 63.3 | 79.8 | 13.5 | 4.5 | 317.4 | 22.7 |
| 9 | 329.0 | 80.4 | 85.2 | 20.8 | 14.4 | 104.8 | 24.8 |
| 12 | 101.4 | 97.9 | 86.0 | 24.5 | 6.0 | 100.0 | 46.3 |
| 15 | 110.6 | 109.4 | 88.6 | 27.6 | 4.3 | 99.6 | 59.1 |
| 18 | 112.4 | 126.2 | 92.1 | 30.6 | 3.5 | 340.4 | 57.6 |
| 21 | 106.1 | 153.1 | 86.3 | 35.0 | 3.5 | 218.5 | 53.4 |
| 24 | 353.7 | 171.8 | 84.1 | 37.7 | 3.4 | 139.3 | 56.0 |
| 27 | 269.7 | 194.3 | 86.7 | 31.4 | 3.7 | 259.8 | 50.1 |
| 30 | 243.2 | 154.9 | 98.9 | 26.7 | 3.9 | 315.4 | 50.8 |
|  |  |  |  |  |  |  |  |
|  | Summer | | | | | | |
| 3 | 150.4 | 103.7 | 92.2 | 4.9 | 6.3 | 62.6 | 46.6 |
| 6 | 224.1 | 148.8 | 107.1 | 33.5 | 25.4 | 94.0 | 39.3 |
| 9 | 298.8 | 143.2 | 111.6 | 45.5 | 39.6 | 124.4 | 50.1 |
| 12 | 352.6 | 160.2 | 105.8 | 60.0 | 55.9 | 187.8 | 50.7 |
| 15 | 243.1 | 162.4 | 133.3 | 60.6 | 58.0 | 188.2 | 45.8 |
| 18 | 103.3 | 168.9 | 124.1 | 56.7 | 61.9 | 166.5 | 58.6 |
| 21 | 217.2 | 174.8 | 130.7 | 66.9 | 70.0 | 160.7 | 49.9 |
| 24 | 293.9 | 169.1 | 117.6 | 69.2 | 67.6 | 199.7 | 52.6 |
| 27 | 384.2 | 181.8 | 140.8 | 70.3 | 58.7 | 192.9 | 49.8 |
| 30 | 127.8 | 150.4 | 144.2 | 69.1 | 76.3 | na | 49.9 |
